# Case Report: Disposition of Symptomatic Probable COVID-19

**DOI:** 10.5811/cpcem.2020.5.48318

**Published:** 2020-06-05

**Authors:** Aleq Jaffery, John Slakey, David Zodda, Douglas Finefrock

**Affiliations:** * Hackensack University Medical Center, Department of Emergency Medicine, Hackensack, New Jersey; † Hackensack Meridian School of Medicine at Seton Hall University, Department of Emergency Medicine, Hackensack, New Jersey

**Keywords:** COVID-19, coronavirus, pneumonia, disposition, MulBSTA

## Abstract

**Introduction:**

The novel coronavirus disease 2019 (COVID-19) presents a challenge for healthcare providers in terms of diagnosis, management, and triage of cases requiring admission.

**Case Report:**

A 47-year-old male with symptoms suspicious for COVID-19, pulse oximetry of 93% on room air, and multifocal pneumonia was risk stratified and safely discharged from the emergency department (ED) despite having moderate risk of progression to acute respiratory distress syndrome. He had resolution of his symptoms verified by telephone follow-up.

**Conclusion:**

Various risk-stratifying tools and techniques can aid clinicians in identifying COVID-19 patients who can be safely discharged from the ED.

## INTRODUCTION

An emerging challenge in the management of patients with coronavirus disease 2019 (COVID-19) symptoms involves the disposition of those with moderate risk for decompensation. Clinicians must grapple with the desire to be conservative with this novel disease entity and the bitter truth that hospital beds as well as life-saving resources are increasingly limited. A growing body of evidence has identified key clinical factors associated with increased morbidity for these patients (Table). There are in addition many clinical tools available to aid in the diagnosis and disposition of these patients. While data are still emerging and not all the tools are fully validated, the growing corpus of evidence as well as shared decision-making and ensuring follow-up are essential parts of safely dispositioning moderate-risk patients. Here we present a case of a 47-year-old male with symptoms suspicious for COVID-19 and with moderate risk of progression to acute respiratory distress syndrome (ARDS) who was discharged home from our emergency department (ED). We discuss which factors contributed to a favorable outcome and how to apply this to patients on a larger scale.

## CASE REPORT

In March 2020, a 47-year-old man presented to a suburban New Jersey hospital at the national epicenter of the COVID-19 pandemic. The patient had no past medical history of known illness. He presented with a chief complaint of persistent fever for 14 days as well as productive cough, scant hemoptysis, sore throat, generalized body aches, and worsening shortness of breath. He was seen by his primary care provider and prescribed a five-day course of amoxicillin. His symptoms did not resolve, so he presented to the ED. He denied recent travel, exposure to positive COVID-19 patients, and any history of smoking.

The patient’s vital signs included a temperature of 38.3° Celsius, a heart rate of 110 beats per minute, a blood pressure of 103/62 millimeters of mercury, and respiratory rate of 21 breaths per minute. His oxygen saturation was 93% on room air. He was overweight with a body mass index of 29.1 kilograms (kg) per meter (m)^2^ (normal range 18.5–24.9 kg/m^2^). His physical exam was otherwise benign including a pulmonary exam with clear and equal breath sounds. Despite tachypnea, he showed no signs of respiratory distress. He additionally had no signs of dehydration, cardiovascular collapse, or rash.

The leading diagnosis was for COVID-19 or another respiratory virus given the geographical area and timing of his presentation, and the patient received a chest radiograph (CXR), a COVID-19 test, and a respiratory pathogen panel (RPP). His RPP was negative for a host of common viruses, and the COVID-19 test had a processing time of several days. His CXR showed multifocal, patchy, airspace opacities at the bilateral lower lobes concerning for multifocal infectious pneumonitis (Image).

Given his oxygen saturation and his CXR, the team discussed with the patient and his wife his risk factors and the likelihood of progression to ARDS. At this point, the patient did not require supplemental oxygen. The team used shared decision-making and explained the risks of progression of his pneumonitis and the likelihood of COVID-19 infection, as well as the benefits of returning home and avoiding hospitalization. The patient and his wife were given strict return precautions for worsening symptoms, particularly worsening dyspnea, and quarantine instructions and precautions were reviewed at length.^1^ He was discharged home that day with azithromycin and an albuterol metered-dose inhaler. Telephone follow-up was made by the same ED provider to check on the patient, who confirmed his symptoms had abated.

**CPC-EM Capsule**

What do we already know about this clinical entity?

The novel coronavirus disease 2019 (COVID-19) is a rapidly evolving clinical entity that causes a variety of pulmonary and inflammatory conditions of varying severity.

What makes this presentation of disease reportable?

This case involves a patient suspicious for novel coronavirus and provides examples of clinical scoring systems and tools that assisted in a safe disposition.

What is the major learning point?

Not every suspected COVID-19 patient who may decompensate need be admitted. Clinicians have a variety of tools that can assist in decision-making.

How might this improve emergency medicine practice?

Clinicians should use both their acumen and gestalt as well as evidence-based clinical tools in their management and disposition of COVID-19 patients.

## DISCUSSION

An important step for clinicians treating confirmed or suspected COVID-19 patients is to risk stratify them and generate a clinical picture with probability of illness progression. In terms of risk, this patient was under 65 years old with no medical comorbidities, two of the most important predictors of morbidity in COVID-19.^2^,^4^ Had his oxygen saturation been lower, or had there been signs of respiratory distress on physical examination, more laboratory values may have been obtained. Laboratory values associated with increased morbidity and mortality from COVID-19 include lymphopenia and elevated acute phase reactants (Table).^2^,^3^,^4^,^5^ These, however, are not necessarily always indicated in the acute setting.

His RPP was of limited clinical utility given the possibility of coinfection;^5^ however, his CXR was concerning, given that bilateral interstitial infiltrates are a common finding in COVID-19 patients.^3^,^4^,^5^

After information-gathering, clinicians can combine the clinical picture with a variety of clinical tools. In critically ill patients with COVID-19 pneumonia, the sequential organ failure assessment score has been shown to correlate with mortality.^6^ The pneumonia severity index (PSI) has traditionally been used to predict risk in patients with community-acquired pneumonia, although it has not been exclusively validated for COVID-19 pneumonia.^7^ Alternatively, a yet-to-be validated tool, initially developed for risk stratifying viral pneumonia in China, shows promise for application in COVID patients. It evaluates **M**ultilobular infiltration, **L**ymphocytopenia, **B**acterial coinfection, **S**moking history, hyper**T**ension, and **A**ge greater than or equal to 60 into the MuLBSTA score, an acronym of its composing parts.^8^ It can also be calculated with less information than the more detailed PSI, although it still requires a complete blood count to evaluate for lymphopenia. Each individual point on the score corresponds to a different 90-day mortality value. The cutoff between low-risk patients and high-risk patients is a score of 12, which corresponds to a 90-day mortality of 16%. Had this patient had a normal white blood cell count, he would have received five points on his MuLBSTA score, corresponding to a 90-day mortality of 2.17%, which is rather high for a “low-risk” designation. Nevertheless, this tool may have helped reinforce what the physical exam, vital signs, and CXR already revealed: that this patient may yet deteriorate and require respiratory support.

Finally, what is the disposition? Should we admit or discharge? This often involves more gestalt than clinical science. The patient was a good candidate for discharge given his likelihood of convalescing and healing, his ability to understand the risks of discharge, his ability to return should symptoms progress, his social network which was capable of offering him support and, most importantly, the ability of the emergency physician to contact him and follow up. It is also essential that this decision be shared between the clinician and the patient and that the clinician stresses the risks and benefits of hospitalization. Ultimately, the shared conversation and education of the patient and the ability to follow-up are the most essential factors in discharging moderate risk patients.

## CONCLUSION

The patient in our case had moderate-risk COVID-19 but had resolution of symptoms without the need for supplemental oxygen therapy or hospital admission. Key contributing factors to positive outcomes include strict return precautions and a follow-up plan. However, not all cases of COVID-19 will resolve. Patients may return at the threshold of intubation, or with new, unpredicted clinical manifestations of this novel virus. Clinicians must be prepared to accept a degree of uncertainty with the risk of a bounceback return to the ED. These risks should be discussed with patients and their preferred support system. In the setting of a global pandemic where ventilators and beds are numbered, a bounceback should not be viewed as a failure of care. Instead, thoughtful disposition of moderate-risk COVID-19 discharges should be seen as temporizing measures to best use our resources. In a practical setting, this may mean the difference between life or death for a patient.

## Figures and Tables

**Image:**
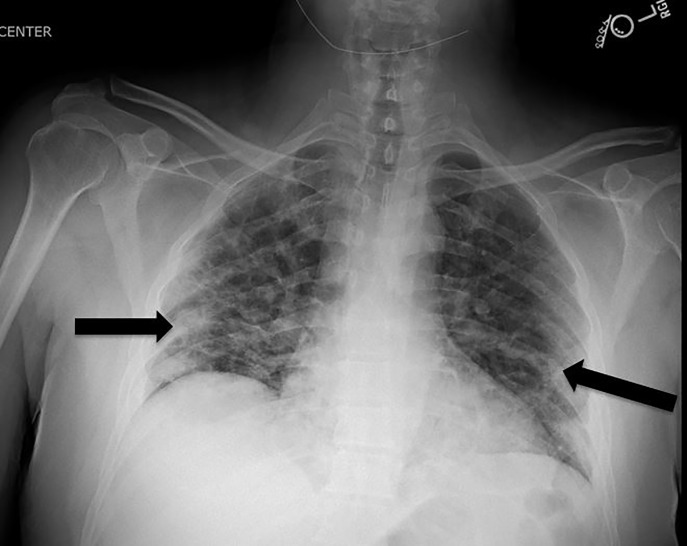
Chest radiograph of the patient. Note bilateral patchy opacities, most prominently at periphery of the lung concerning for multifocal pneumonia (arrows).

**Table:** Factors identified with morbidity and mortality in novel coronavirus disease 2019 patients.^2^,^3^,^4^,^5^

Age > 65 Comorbid conditions, i.e., diabetes mellitus, hypertension Lymphopenia Elevated D-dimer Elevated lactate dehydrogenase Elevated C-reactice protein Elevated erythrocyte sedimentation rate Elevated ferritin Low albumin
